# Weight-for-age distributions among children with HIV on antiretroviral therapy in the International epidemiology Databases to Evaluate AIDS (IeDEA) multiregional consortium

**DOI:** 10.1186/s13104-020-05081-7

**Published:** 2020-05-24

**Authors:** Julie Jesson, Sophie Desmonde, Constantin T. Yiannoutsos, Gabriela Patten, Karen Malateste, Stephany N. Duda, Nagalingeswaran Kumarasamy, Marcel Yotebieng, Mary-Ann Davies, Beverly Musick, Valeriane Leroy, Andrea Ciaranello, IeDEA

**Affiliations:** 1 Inserm U1027, Université Paul Sabatier Toulouse 3, Toulouse, France; 2 Indiana University, Fairbanks School of Public Health, Indianapolis, IN USA; 3 School of Public Health and Family Medicine, Faculty of Health Sciences, University of Cape Town, Cape Town, South Africa; 4 Inserm U1219, Bordeaux, France; 5 Bordeaux Population Health Center, Université de Bordeaux, Bordeaux, France; 6 Vanderbilt University Medical Center, Nashville, TN USA; 7 Chennai Antiviral Research and Treatment Clinical Research Site (CART CRS), VHS-Infectious Diseases Medical Centre, VHS, Chennai, India; 8 Division of Epidemiology, The Ohio State University, College of Public Health, Columbus, OH USA; 9 Indiana University School of Medicine, Indianapolis, IN USA; 10 The Medical Practice Evaluation Center, Boston, MA USA; 11 The Division of General Internal Medicine, Department of Medicine, Harvard Medical School, Boston, MA USA; 12 Massachusetts General Hospital, Boston, MA USA; 13 Inserm, UMR 1027 - Epidémiologie et analyses en santé publique: risques, maladies chroniques et handicaps - Université Paul Sabatier Toulouse 3, Equipe 2: Axe santé de l’enfant et de l’adolescent en Afrique, 37 Allées Jules Guesde, 31073 Toulouse Cedex 7, France, Toulouse Cedex 7, France

**Keywords:** Pediatric HIV, Pediatric antiretroviral therapy, Antiretroviral formulation, Weight-for-age

## Abstract

**Objective:**

Pediatric antiretroviral therapy (ART) for children with HIV (CHIV) must be dosed appropriately for children’s changing weights as they grow. To inform accurate estimates of ART formulations and doses needed, we described weight-for-age distributions among CHIV on ART in the IeDEA global pediatric collaboration between 2004 and 2016, using data from six regions (East, West, Central, and Southern Africa, Asia–Pacific, and Central/South America and the Caribbean).

**Results:**

Overall, 59,862 children contributed to the analysis. Age and weight data were available from 530,080 clinical encounters for girls and 537,894 for boys. For each one-year age stratum from 0 to 15 years, we calculated the proportion of children in each of the weight bands designated by the World Health Organization as relevant to pediatric ART formulations: 0 to  < 3 kg, 3 to  < 6 kg, 6 to  < 10 kg, 10 to  < 14 kg, 14 to  < 20 kg, 20 to  < 25 kg, 25 to  < 30 kg, 30 to  < 35 kg, 35 to  < 40 kg, 40 to  < 45 kg, 45 to  < 50 kg, 50 to  < 55 kg, 55 to  < 60 kg, and ≥ 60 kg. Data are reported for the entire cohort, as well as stratified by sex and IeDEA region, calendar year of ART use, and duration on ART at time of assessment (< 12 or ≥ 12 months), provided in data tables. These data are critical to improve the accuracy of forecasting and procurement of pediatric ART formulations as the pediatric HIV epidemic and pediatric HIV treatment strategies evolve.

## Introduction

Children with HIV (CHIV) require appropriate dosing of pediatric antiretroviral therapy (ART) to prevent HIV-related morbidity and mortality. HIV programs at national and international levels procure annual quantities of pediatric ART based on forecasts of the number of children likely to require each drug formulation and dose; dosing is weight-based in children < 15 years of age. Accurate forecasting of antiretroviral needs is particularly important, because the pediatric antiretroviral market is small. Procurement volume has a large impact on market prices and failure to procure sufficient quantities of antiretroviral medications (ARVs) leads to gaps in treatment access in low-income and middle-income countries [1]. While validated estimates exist of the numbers of children at each age likely to need ART [2], additional information about the weight distribution for CHIV by age are needed to permit accurate forecasts of the medication quantities that will be required. To date, ART forecasts rely on WHO and CDC growth curves for all children in the general population [3–5]. However, children and adolescents living with HIV are often malnourished at the time of initiating ART and their catch-up growth can be delayed even after initiating ART [6–9]. Because of these key differences for CHIV, growth curves derived from the general population likely overestimate weight-for-age among CHIV and therefore may lead to inaccurate estimates of the number of formulations and doses of pediatric ART that will be required globally [10]. Accurate data about volume trends in weight-for-age evolution among ART-treated CHIV are therefore needed to appropriately inform ART forecasting efforts [11].

This study reports a secondary analysis of data from a parent study conducted within the global International epidemiology Databases to Evaluate AIDS (IeDEA) pediatric research consortium (https://www.iedea.org/). The parent study sought to analyze age- and CD4-stratified risks of opportunistic infections and mortality for CHIV before and after ART initiation in six global IeDEA regions [12]. Using the database assembled for the parent study, we conducted a separate analysis, reported here, to evaluate the weight-for-age distributions in CHIV on ART in the IeDEA consortium in order to support accurate forecasting and procurement of pediatric ART formulations.

## Main text

### Methods

We analyzed individual patient data from the six regional pediatric cohorts within IeDEA: Asia–Pacific, West Africa, East Africa, Central Africa, Southern Africa, and the Caribbean, Central, and South America network (CCASAnet) [13]. Using eligibility criteria from the parent study [12], all patients were included if they enrolled into care at age < 24 years, were followed-up at any of the participating IeDEA sites between 2004 and 2016, had a confirmed HIV diagnosis, were ART-naïve at enrolment, and had at least one CD4 count or percent measurement during follow-up. In the current analysis, we limited the dataset to children aged < 15 years, because older youth primarily use adult ARV formulations and doses. Although clinic protocols vary across the IeDEA consortium, CHIV are usually seen at least every 3 months while on ART. The data were generated during routine care encounters and included region, country, site, patient demographics (sex, date of birth, date of HIV diagnosis if available, and date of enrolment in care), laboratory values (CD4 count, CD4 percent), date of ART initiation, initial ART regimen, date of death, date of last clinical contact, and date of transfer out. Each participating IeDEA region obtained local institutional review board approval to participate. Written informed consent requirements were deferred to the local institutional review boards. The analysis only used de-identified data that had been collected as part of routine clinical care.

For the current study, we used data on weight at each visit as recorded in the medical record. For each one-year age stratum from 0 to 15 years, we calculated the number of children in each of the weight bands designated by the World Health Organization (WHO) to be relevant to pediatric ART formulations: 0 to  < 3 kg, 3 to  < 6 kg, 6 to  < 10 kg, 10 to  < 14 kg, 14 to  < 20 kg, 20 to  < 25 kg, 25 to  < 30 kg, 30 to  < 35 kg, 35 to  < 40 kg, 40 to  < 45 kg, 45 to  < 50 kg, 50 to  < 55 kg, 55 to  < 60 kg, and ≥ 60 kg. We derived these numbers, and resulting proportions, for the entire cohort of CHIV, total and stratified by sex; we additionally derived numbers and proportions stratified by sex and IeDEA region, calendar year of enrolment (before versus beyond January 1, 2013), and time on ART (< 12 months or ≥ 12 months). Children for whom sex was unknown were excluded from the analysis. Age at each visit was calculated based on the date of birth recorded in the database. A single child contributed multiple weight measurements over time and we deleted outlying measurements based on the following criteria: (1) weight-for-age z-scores (WAZ) > 3 for infants aged < 1 year, WAZ < − 8 for children aged < 5 years, WAZ < − 6 for children aged 5–10 years, and WAZ > 5 for all ages; (2) two values measured within the same month with a difference greater than 5 kg if age < 5 years, 8 kg if age 5 to  < 10 years, and 10 kg if age 10–15 years.

### Results

Overall, 59,862 children and adolescents with HIV contributed to the analysis, of whom 50.7% were females. Demographic information is shown in Table 1. Age and weight data were available from 530,080 clinical encounters for girls and 537,894 clinical encounters for boys. Sex-stratified results for all CHIV in the study are shown in Table 2. Additional tables are included in Additional file 1:

1. Overall population (combined).
2. By sex (Table 2).
3. By IeDEA region, boys.
4. By IeDEA region, girls.
5. By ART duration and sex.
6. By year of visit and sex.
7. By IeDEA region, ART duration, boys.
8. By IeDEA region, ART duration, girls.
9. By IeDEA region, ART duration, < 2013, Boys.
10. By IeDEA region, ART duration, < 2013, Girls.
11. By IeDEA region, ART duration, ≥ 2013, Boys.
12. By IeDEA region, ART duration, ≥2013, Girls.

**Table 1:** Characteristics of the 59,862 children in IeDEA at baseline

Characteristic	
Region, n (%)	
Asia–Pacific	4004 (6.7)
CCASAnet	1502 (2.5)
Central Africa	2050 (3.4)
East Africa	11,068 (18.5)
Southern Africa	36,679 (61.3)
West Africa	4559 (7.6)
Age, median (IQR), years	6.1 (2.5–9.9)
Female, n (%)	30,343 (50.7)
Year at ART initiation	
Year, n (%)	
< 2004	1729 (2.9)
2004–2007	17,821 (29.8)
2008–2012	31,272 (52.2)
2013–2014	7493 (12.5)
2015–2016	1547 (2.6)
Number of observations by year of visit	
< 2004	16,505 (1.5)
2004–2007	178,985 (16.8)
2008–2012	610,078 (57.1)
2013–2014	207,899 (19.5)
2015–2016	54,507 (5.1)

*CCASAnet* the Caribbean, Central and South American Network, *ART* antiretroviral therapy, *IQR* interquartile range

**Table 2:** Weight-for-age distribution of CHIV aged 0–15 in the IeDEA global consortium (N = 59,862 children from 2004 to 2016)

Weight bands (kg)	Age (years)—boys—537,894 observations																
	0.25	0.75	1	2	3	4	5	6	7	8	9	10	11	12	13	14	15
00–03	1.029	0.028	0.000	0.000	0.000	0.000	0.000	0.000	0.000	0.000	0.000	0.000	0.000	0.000	0.000	0.000	0.000
03–06	40.403	13.020	2.783	0.292	0.021	0.013	0.000	0.000	0.000	0.000	0.000	0.000	0.000	0.000	0.000	0.000	0.000
06–10	55.844	77.778	54.134	14.926	2.702	0.706	0.251	0.056	0.000	0.000	0.000	0.000	0.000	0.000	0.000	0.000	0.000
10–14	2.724	9.174	41.096	69.703	48.974	22.369	8.940	3.514	1.437	0.589	0.232	0.222	0.133	0.112	0.148	0.088	0.033
14–20	0.000	0.000	1.976	14.846	47.473	72.577	76.439	61.465	39.180	20.047	9.155	4.328	2.015	1.234	0.641	0.573	0.386
20–25	0.000	0.000	0.010	0.233	0.695	3.975	13.351	31.628	49.521	55.249	46.544	30.485	18.679	10.502	5.967	3.306	1.796
25–30	0.000	0.000	0.000	0.000	0.136	0.331	0.847	2.794	8.650	20.675	34.73	43.902	42.407	33.264	21.418	13.45	7.993
30–35	0.000	0.000	0.000	0.000	0.000	0.029	0.133	0.393	0.827	2.66	7.422	16.387	26.234	33.233	34.623	26.571	19.027
35–40	0.000	0.000	0.000	0.000	0.000	0.000	0.039	0.138	0.277	0.517	1.29	3.289	7.68	14.467	20.806	24.61	21.694
40–45	0.000	0.000	0.000	0.000	0.000	0.000	0.000	0.012	0.051	0.165	0.34	0.777	1.63	4.711	9.329	16.108	19.315
45–50	0.000	0.000	0.000	0.000	0.000	0.000	0.000	0.000	0.037	0.049	0.129	0.287	0.65	1.334	4.072	8.19	14.515
50–55	0.000	0.000	0.000	0.000	0.000	0.000	0.000	0.000	0.021	0.026	0.062	0.15	0.289	0.571	1.529	4.099	8.445
55–60	0.000	0.000	0.000	0.000	0.000	0.000	0.000	0.000	0.000	0.021	0.036	0.065	0.133	0.304	0.787	1.61	4.007
60+	0.000	0.000	0.000	0.000	0.000	0.000	0.000	0.000	0.000	0.002	0.06	0.107	0.149	0.267	0.679	1.395	2.790

Weight bands (kg)	Age (years)—girls—530,080 observations																
	0.25	0.75	1	2	3	4	5	6	7	8	9	10	11	12	13	14	15
00–03	1.450	0.000	0.010	0.000	0.000	0.000	0.000	0.000	0.000	0.000	0.000	0.000	0.000	0.000	0.000	0.000	0.000
03–06	47.228	17.665	3.921	0.321	0.091	0.034	0.000	0.000	0.000	0.000	0.000	0.000	0.000	0.000	0.000	0.000	0.000
06–10	50.127	75.964	62.114	19.880	3.904	0.881	0.315	0.123	0.048	0.007	0.000	0.000	0.000	0.000	0.000	0.000	0.000
10–14	1.195	6.371	32.565	69.966	56.795	28.381	11.887	5.035	2.146	0.921	0.329	0.217	0.130	0.178	0.058	0.087	0.031
14–20	0.000	0.000	1.390	9.630	38.328	67.443	76.477	65.108	43.060	23.990	11.517	4.638	1.986	0.984	0.624	0.362	0.252
20–25	0.000	0.000	0.000	0.199	0.746	2.968	10.430	27.026	46.218	53.359	46.154	31.204	17.168	8.219	3.893	1.823	0.954
25–30	0.000	0.000	0.000	0.004	0.120	0.233	0.727	2.299	7.298	18.147	32.015	39.685	35.326	24.874	14.544	7.609	3.890
30–35	0.000	0.000	0.000	0.000	0.016	0.050	0.087	0.270	0.880	2.693	7.473	16.964	26.575	28.392	23.444	16.438	10.333
35–40	0.000	0.000	0.000	0.000	0.000	0.011	0.061	0.084	0.208	0.562	1.705	4.924	11.608	19.555	22.452	21.150	16.452
40–45	0.000	0.000	0.000	0.000	0.000	0.000	0.015	0.037	0.074	0.140	0.444	1.525	4.618	10.380	17.761	21.965	22.354
45–50	0.000	0.000	0.000	0.000	0.000	0.000	0.000	0.020	0.029	0.071	0.146	0.436	1.663	4.346	9.546	15.818	20.048
50–55	0.000	0.000	0.000	0.000	0.000	0.000	0.000	0.000	0.026	0.071	0.102	0.125	0.445	1.884	4.505	8.199	13.734
55–60	0.000	0.000	0.000	0.000	0.000	0.000	0.000	0.000	0.012	0.021	0.066	0.117	0.199	0.623	1.854	3.798	6.581
60+	0.000	0.000	0.000	0.000	0.000	0.000	0.000	0.000	0.000	0.017	0.049	0.164	0.283	0.564	1.321	2.752	5.371

## Limitations

This study is subject to a number of limitations. Several characteristics of patients participating in the IeDEA cohort may lead to overestimation of the weight-for-age distribution of children receiving ART and thus may overestimate the amount of active pharmaceutical ingredient required per child. The first is survivorship bias, because CHIV who survived to initiate ART at IeDEA sites from 2004 to 2016 may have been healthier (and perhaps with higher weights for age) than those who did not. Children in this cohort do not represent a complete cohort of CHIV followed from birth, and there was likely a high risk of death in children before the age of 2 years who therefore never entered the study. Similarly, a high proportion of CHIV were lost to follow-up during the study period, possibly leaving a healthier cohort to remain in care and with evaluable weights and ages. Nevertheless, these data likely accurately reflect weight-for-age among children who remained on treatment and for whom ARV procurement estimates currently apply, even if they may overestimate weight for the full population of CHIV in need of ART. On the other hand, approximately two-thirds of IeDEA sites are located at university hospitals in capital cities, where the standard of care may be different than in rural areas; if more intensive HIV care is provided, children at IeDEA sites may be healthier than at more rural sites in the same regions [13]. We also note a potential selection bias induced by the parent study, which only included children with available CD4 counts during follow-up, thus excluding the sicker children who may have initiated ART immediately, without their caregivers waiting for CD4 counts. This may also be possibly overestimating weights for ART-treated CHIV.

A second set of limitations may counteract these trends and may underestimate the quantities of active pharmaceutical ingredients needed. For example, before ART was recommended for all CHIV in 2013, it was offered only to the sickest children, potentially introducing an indication bias. This selection bias may particularly apply to older children in the IeDEA cohort, as access to early ART has improved over time for children, so adolescents aged 10–15 years in the future may be in better health than those observed in the IeDEA cohort over the study period reported in this study [14].

Despite these limitations, this dataset is, to our knowledge, the only available source of data about weight-for-age distributions among CHIV treated with ART built on the strength of a large cohort size and broad geographic coverage. Consequently, the resulting estimates and those, more generally, derived from observational cohorts conducted in real-world settings, offer a valuable opportunity to derive data-driven weight-for-age distributions among CHIV [15, 16].

These data were originally pooled to support adequate forecasting and procurement of pediatric ART formulations. Nevertheless, beyond this purpose, data about weight in children may also be helpful to create a secondary-hand framework for observing changes overtime regarding growth on ART, during childhood and adolescence as this has been done in the past [7, 11]. Especially, these data will be useful in refining projections of the number of CHIV who may need specific ARV formulations and doses allowing HIV care and treatment programs to procure sufficient quantities of medications for CHIV, avoiding both medication stock outs and medication wastage, furthering the critical goal of improving access to ART for CHIV globally [1].

## Supplementary information

**Additional file 1.** Weight-for-age distributions among CHIV on ART in the International Epidemiology to evaluate AIDS (IeDEA) multiregional consortium - complete tables.

## Data Availability

The datasets generated during and/or analyzed during the current study are not publicly available due to legal and ethics restrictions. The principles of collaboration of the IeDEA cohort consortium and the regulatory requirements of the individual member site and country institutional review boards require the submission and approval of a project concept sheet by the IeDEA Executive Committee, and the principal investigators and local site investigators from participating regions. IeDEA promotes the signing of a Data Use Agreement before HIV clinical data can be released. Individuals interested in obtaining access to data may contact IeDEA for additional information at https://www.iedea.org/home/who-we-are/. The project concept sheet template and other research-related resources are available at https://www.iedea.org/resources/.

## Abbreviations

CHIV: Children with HIV
ART: Antiretroviral Therapy
ARV: Antiretroviral medication
IeDEA: International epidemiology Databases to Evaluate AIDS
WHO: World Health Organization
